# Design and Validation of Miniaturized Repetitive Transcranial Magnetic Stimulation (rTMS) Head Coils

**DOI:** 10.3390/s24051584

**Published:** 2024-02-29

**Authors:** Shaghayegh Abbasi, Sravya Alluri, Vincent Leung, Peter Asbeck, Milan T. Makale

**Affiliations:** 1 Electrical Engineering Department, University of Portland, Portland, OR 97203, USA; abbasi@up.edu; 2 Department of Electrical and Computer Engineering, University of California San Diego, La Jolla, CA 92093, USA; 3 Calit2 Advanced Circuits Laboratory, University of California San Diego, La Jolla, CA 92093, USA; 4 Department of Electrical and Computer Engineering, Baylor University, Waco, TX 76706, USA; 5 Moores Cancer Center, Department of Radiation Medicine and Applied Sciences, University of California San Diego, La Jolla, CA 92093, USA

**Keywords:** figure-8 coil, pulsed magnetic field, brain stimulation, miniaturized rTMS system

## Abstract

Repetitive transcranial magnetic stimulation (rTMS) is a rapidly developing therapeutic modality for the safe and effective treatment of neuropsychiatric disorders. However, clinical rTMS driving systems and head coils are large, heavy, and expensive, so miniaturized, affordable rTMS devices may facilitate treatment access for patients at home, in underserved areas, in field and mobile hospitals, on ships and submarines, and in space. The central component of a portable rTMS system is a miniaturized, lightweight coil. Such a coil, when mated to lightweight driving circuits, must be able to induce B and E fields of sufficient intensity for medical use. This paper newly identifies and validates salient theoretical considerations specific to the dimensional scaling and miniaturization of coil geometries, particularly figure-8 coils, and delineates novel, key design criteria. In this context, the essential requirement of matching coil inductance with the characteristic resistance of the driver switches is highlighted. Computer simulations predicted E- and B-fields which were validated via benchtop experiments. Using a miniaturized coil with dimensions of 76 mm × 38 mm and weighing only 12.6 g, the peak E-field was 87 V/m at a distance of 1.5 cm. Practical considerations limited the maximum voltage and current to 350 V and 3.1 kA, respectively; nonetheless, this peak E-field value was well within the intensity range, 60–120 V/m, generally held to be therapeutically relevant. The presented parameters and results delineate coil and circuit guidelines for a future miniaturized, power-scalable rTMS system able to generate pulsed E-fields of sufficient amplitude for potential clinical use.

## 1. Introduction

Repetitive transcranial magnetic stimulation (rTMS) has rapidly expanded as a safe and effective therapeutic intervention for treatment-resistant depression and anxiety, as well as for beneficially modulating neuronal activity in the brain’s cortical regions [1,2,3,4,5,6,7,8]. Devices able to deliver rTMS are used externally, placed against the scalp, to generate a magnetic field that penetrates the brain cortex and induces an electric field that exerts neurophysiological effects. Current rTMS clinical systems are large, heavy, complex, and costly, so disadvantaged areas and populations, as well as small medical facilities and environments, may have inadequate rTMS availability [9]. Moreover, many patients are unable to visit a standard rTMS clinic more than two or three times weekly, and currently, most patients are not treated on weekends. Patients with major depressive disorder (MDD) often cannot or will not come to an rTMS clinic for treatment, especially twice a day, which studies have shown is efficacious [10]. Hence, an emergent view from many mental health professionals is that a viable solution may be inexpensive, portable home-use rTMS devices, particularly as post-COVID demand for telemedicine and home treatment for mental health disorders has remained high [11,12,13,14,15,16,17]. A recent German study found that 50% of people would prefer mental health treatment at home, and another 20% indicated preference for online therapy [18]. A Swiss study demonstrated that even mental health crisis resolution treatment at home was as effective as standard hospitalization in terms of reduction in symptoms, rates, and durations of readmissions, as well as treatment length [19]. Lee and colleagues (2019) discussed and attempted to pursue miniaturized rTMS for home treatment [12]. Hence, a miniaturized, portable, affordable, and lightweight rTMS system may expand access to a wide array of subjects. It may also facilitate the realization of next-generation rTMS, including multi-coil arrays and possible integration with a scaled-down EEG acquisition array and controller to form a closed-loop therapeutic system [20,21].

The concept of a portable/wearable rTMS device has been previously proposed [22,23,24], but a sufficiently miniaturized system adaptable to rechargeable, ambulatory use, and able to repetitively and adequately stimulate the human brain cortex, is a technically challenging proposition. This stems from the demands of high voltage and current levels, rapid magnetic pulse rise times, and stringent safety regulations [25]. A key component of an rTMS system is the head coil, which must be able to provide therapeutically adequate magnetic (B) fields and electric (E) fields. Accordingly, the creation of a small, lightweight head coil must meet a specific design framework, and its design properties dictate the topology of the companion electronic driver circuits.

The present study is novel in that it blends theoretical considerations, modeling, and experimentation to identify and validate key scaling and design criteria for a miniaturized rTMS system that could at least approach clinically relevant E-field strengths. Moreover, the E-fields we generated align with E-field strengths that reports indicate are therapeutically relevant [26,27,28]. With the present study, we have embarked on a research and development effort towards a field-testable, miniaturized rTMS prototype. Human testing was beyond the intended scope of this work, although follow-up studies and reports will certainly include human tests.

One goal is to adapt the miniaturized rTMS device for ambulatory use to provide frequent treatments, worn under a cap, hat, or bandana, for example. In this context, a coil weighing 12.6 g is much more feasible than one weighing 1–3 kg. A miniaturized coil has higher focality compared to standard set up coils, making it possible to target specific brain regions with higher spatial resolution, which has been shown to increase the effectiveness of treatment [29,30,31,32]. Moreover, an array of several small, lightweight coils may focus B- and E-fields to multiple specific locations in the cortex, facilitating personalized, precise treatment [20,33,34].

Another important factor supporting the development of a miniaturized system is affordability. We have costed our miniaturized prototype to about 1/10th of even a moderately priced full-scale clinical system. While miniaturization does not always translate to lower cost, in this instance, smaller size means fewer materials and smaller, less costly power systems and switching components. Our prototype required a power supply voltage of only 350 V as opposed to 1200–3000 V, yet it did generate therapeutically relevant fields. The prototype system described here is the first of its kind, and has essentially attained the goal of generating therapeutic E-field strengths in a miniaturized platform.

Magnetic field intensities applied in clinical rTMS systems are generally in the range of 0.3–2 Tesla (T), measured at the scalp (bottom surface of the coil) [28,35]. Brain cortex stimulation in rTMS is generally characterized at 1.5 cm from the surface of the coil, which is placed against the scalp [36,37,38]. At this depth, brain cortical E-fields induced by full-scale commercial rTMS systems in widespread therapeutic use [28] are typically in the range of 60–120 V/m. In representative clinical systems, coils are excited with pulsed currents, either monophasic or biphasic, with duration of 60–280 µs, which are repeated at 10 Hz over 10 s. The resulting pulse trains (of 100 pulse lengths) are repeated on the order of 20 times with a 30 s period. Coil heating must be limited during such a procedure, which establishes constraints for coil electrical and thermal resistances.

In view of the forgoing, the design tradeoffs for an rTMS system with miniaturized coils involve the relationships between system parameters as the coil dimensions are scaled down. As the dimensions of a coil are changed, the associated electromagnetic fields scale in a relatively complex fashion. For example, for a circular coil with radius R, the magnetic field at the center of the coil increases as 1/R as the coil size is reduced. However, at a fixed depth z from the coil, with z ≫ R, the magnetic field decreases according to R^2^. The associated electric field obeys different scaling relationships.

Coil design for rTMS has been the subject of numerous publications [28,39,40,41]. While a wide variety of coil structures and geometries have been investigated, in clinical practice, the figure-8 coil is most prominent. For this well-established coil geometry, this paper focuses on criteria germane to a scaled-down rTMS system, including small size and weight, with characteristics compatible with driving circuits which have limited voltage and current handling capability, yet are able to produce E-fields sufficient to elicit therapeutic stimulation levels in the brain cortex. In the present work, the scaling relationships for the E- and B-fields were calculated explicitly for figure-8 coils in the geometry used for rTMS. Results are initially shown for the idealized case of a current carried in wires of infinitesimal thickness, in a homogenous medium (see Supplementary Materials for further explanation).

Subsequent calculations show the fields obtained with refinements of the geometry, taking into account the finite thickness of the wires used in the coils and realistic head geometry. The impact of the number of turns in the coil was also quantified in relation to the characteristics of the electronic switch used to control the coil current. Thus, in this work, key relationships between coil properties (height, turns, thickness) and electronic switching were identified and detailed, and resultant coil designs were simulated in the context of E-field intensity and geometry. For validation, we designed, fabricated, and tested several different scaled-down figure-8 coils, which led to a miniaturized figure-8 coil that was 78 mm long and weighed only 12.6 g. When driven by 350 V and 3.1 kA, it generated a therapeutically relevant peak E-field of 87 V/m at 1.5 cm from the coil center, and was able to sustain 10 Hz for 5 min (3000 pulses).

Our innovative test system attained clinically feasible E-fields, 87 V/m at 1.5 cm, yet the driving circuit weighed less than 5 lbs (2.26 kg), with a 0.4 ounce (12.6 g) head coil. This is in sharp contrast to current clinical rTMS systems that weigh at least 150 lbs (68 kg), most of them much more, and head coils weigh typically 4 lbs (1.8 kg) or more. Importantly, the 87 V/m E-field that we attained at 1.5 cm was well above the limit of 28 V/m, which is the minimum intensity needed to induce neuronal action potential [42,43].

We believe that this work represents an essential first step in a continuum of research and development leading to miniaturized, clinical rTMS systems. The experimental coil and driving system we developed are much smaller and lighter than representative, widely used commercial coils, and to the best of our knowledge, currently available miniaturized coils are only capable of providing E-fields sufficient for mouse experiments [41,43]. We attained peak E-fields that were neurophysiologically relevant and were sustained at 10 Hz for 5 min (3000 pulses) [26,27,28,42,43]. This device can be used, and the criteria established and experimentally validated here may help to guide the future design, fabrication, and evaluation of miniaturized rTMS systems for possible human clinical use.

## 2. Methods

Simulation Framework. An initial coil design was carried out using an idealized figure-8 coil model to determine electric and magnetic fields as a function of depth, assuming the current was carried in wires of infinitesimal thickness in a homogeneous medium. Subsequent calculations were carried out for specific designs taking explicit account of the full 3-dimensional structure, including wire thickness and a realistic head geometry.

**Figure 1:**
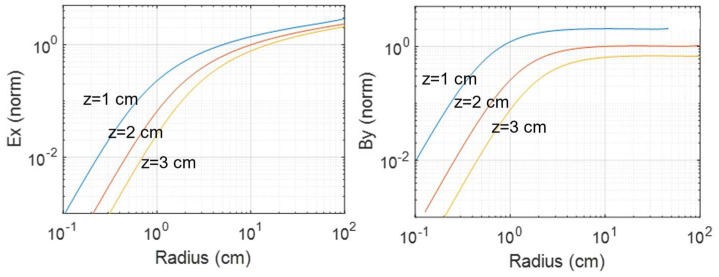
Calculated $E_{x}$ and $B_{y}$ fields vs. depth for figure-8 coils. Fields are normalized to their values at z = 2 cm and R = 10 cm at a given current level.

For the figure-8 coils considered in this work, the scaling relationships for the idealized structure outlined above were determined using the electromagnetic equations given in the Supplementary Materials. In a practical case, the electric field of interest is located under the point of intersection of the two sides of the coil and is oriented in the direction of the current (designated here as the x direction). The associated magnetic field is in the perpendicular y direction. Figure 1 shows the calculated electric field E_x_ and magnetic field B_y_ as a function of coil radius R and depth z from the plane of the coil. The fields were proportional to the current through the coil and number of turns (assumed to be overlapping), and were normalized in the figure to the values obtained at z = 2 cm and R = 10 cm. It was found that, as R was scaled at a fixed current I, the change in the B field was relatively small until R approached z. The scaling of the E field was significantly stronger. For the reduction in R from 10 cm to 1 cm, for example, the B field at a depth of 2 cm dropped by approximately 4× for a fixed current, while the electric field dropped by 16×. Importantly, therefore, system designs to maximize B fields differ from system designs for maximizing E fields. In this context, it must be recognized that, within the patient’s brain, it is the induced E-field that triggers neurophysiological responses to create desired therapeutic effects. A more detailed theoretical discussion is provided in the Supplementary Materials.

Scaling of the fields as the number of turns in the coils (N) is changed is central to the system design. An approximate estimate of the effect of N can be made assuming the wire diameter is negligible (thus all the turns lie in the same plane). As N is varied, the E field and the B field for a fixed current in each loop both scale as N. The overall coil voltage associated with dI/dt during the rTMS pulse scales as N^2^. It is desirable to increase N to maximize the E field at a given z. In the associated electronic circuit design, however, there are typically important constraints on the maximum voltage that can be handled, V_max_, and the maximum current that can be supported, I_max_, along with a target transient pulse duration, ΔT. In the system discussed below, V_max_ is set by the maximum voltage for the IGBT switches, and I_max_ is set by their pulse current handling capability. It is thus necessary to limit N by the following constraint:

(1) $$L\frac{I_{max}}{∆T}<V_{max}$$

where L is the coil inductance and scales as N^2^. With representative switch implementations, there is a characteristic value of V_max_/I_max_, which is defined here as the characteristic resistance R_ch_ of the switch. An important design consideration is to choose N to match L with the switch R_ch_, according to:

(2) $$L=\gamma R_{ch}∆T$$

where γ is a numerical constant of order unity dependent on the current pulse shape, which, for customary systems, as discussed below, has the value 2/π.

The impact of N can be approximately assessed from the fact that the E field at a given depth is proportional to the voltage developed for a single loop (for the case of negligible wire diameter), while the overall coil voltage is N times as large. Thus, for a given constraint on coil voltage V_max_, the maximum E field that can be obtained at the target depth z will be obtained for N = 1, and it will decrease inversely with N.

**Figure 2:**
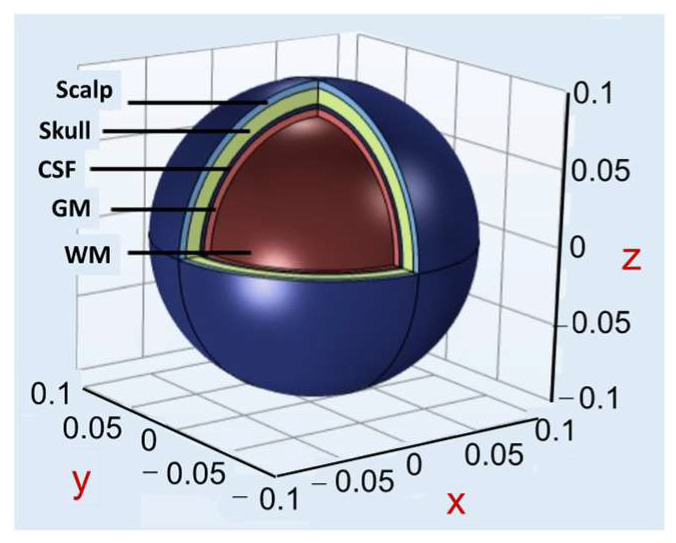
Multilayer spherical head model. Each layer of a realistic human head is modeled here as part of a sphere.

Departures from the simple relationships described above occur due to the finite thickness of the wires used and from the curvature of the head, so more detailed calculations are required. For a more accurate and detailed analysis, a finite element model of the head and the rTMS coil was developed using AC/DC module in COMSOL Multiphysics^®^ [44]. Modeling and simulations were run on a Dell Series 9700 desktop PC computer with a 16 GB hard drive and 8 MB of RAM. The spherical head model had a radius of 10 cm, and consisted of 5 different layers: scalp (skin), skull (bone), cerebral spinal fluid (CSF), gray matter, and white matter (Figure 2), with electrical and magnetic properties derived from the literature [45] (Table 1).

An infinite element domain was used to set the boundary conditions. The spherical head model does not provide precise personalized anatomical fidelity as do 3-D imaging-based models, which recapitulate the sulcal and gyral folding of the cortex, but is generally held to provide broadly relevant results not restricted by individual anatomy. As part of design modifications to achieve a portable system, as well as future integration with EEG electrodes, we quantitatively examined the effect of coil size on the induced electric field using the three following performance metrics:

1. Maximum electric field ($E_{max}$): The maximum E-field created on the surface of the cortex;
2. Half-value depth ($d_{1/2}$): Radial distance from cortical surface to the deepest point inside the cortex where the E-field value is half of its maximum ($E_{max}$);
3. Half-value tangential spread ($S_{1/2}$): This parameter is related to focality of the E-field, and it is defined as follows:

(3) $$S_{1/2}=\frac{V_{1/2}}{d_{1/2}}$$

where $V_{1/2}$ is the half-value volume, defined as the volume of the brain region that is exposed to an electric field stronger than half of the maximum electric field. The lower the value of $S_{1/2}$, the more focal the E-field is [29].

**Figure 3:**
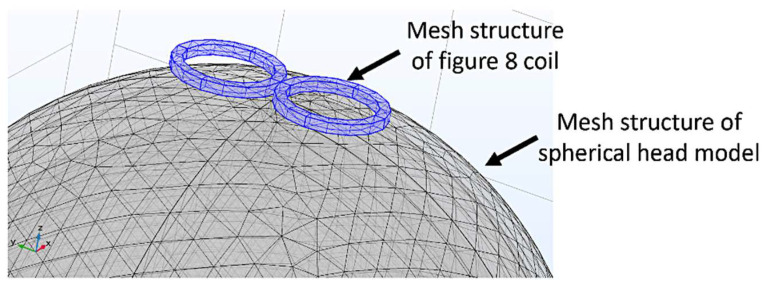
COMSOL mesh structure of a figure-8 rTMS coil on a simplified human head.

The magnetic field strength attainable with compact figure-8 coils was modeled to determine whether small compact coils on the order of 5–15 cm in total length are feasible (Figure 3). A figure-8 coil, which is typically used by most commercial rTMS systems, places 2 circular coils adjacent to each other and uses superposition to create a larger net electric field in the center. This comparatively high degree of focality offers distinct advantages in terms of localizing stimulation to discrete areas of the brain.

The FEM simulations were used to calculate the performance metrics above for figure-8 coils of different sizes. The current pulse characteristics for these simulations were derived from our experimental system as described below. These results were obtained for a monophasic current pulse with a peak value of 3.1 kA and a pulse width of 60 μs. Peak current and pulse width are important design variables that must be traded off for optimal performance. Peak current was limited in our case by the current handling of inexpensive IGBTs, coupled with the fact that heating increases as I^2^. Neurophysiological studies of rTMS have examined pulse widths between 20 and 200 μs, with longer pulses for biphasic waveforms. In order to achieve neuronal activation, the pulse width must be sufficiently long to generate a critical product of electric field amplitude integrated over pulse duration that meets or exceeds the neuronal activation threshold. However, as the pulse duration increases, the energy increases [46,47]. Therefore, we minimized the pulse length to be consistent with the peak current.

Simulation results pertaining to coil size are displayed in Table 2. The size specified for the figure-8 coil was the largest dimension. The number of turns in the coil was 6. The results show how the coil size affected the maximum E-field. It is also evident from this table how the coil size affected the half-value depth ($d_{1/2}$) and half-value tangential spread ($S_{1/2}$), which indicate the depth and focality of the E-field. It is evident from these values that the focality improved as the coil size was reduced, which is one of the main advantages of a miniaturized head coil, others being substantially reduced weight and dimensions.

**Figure 4:**
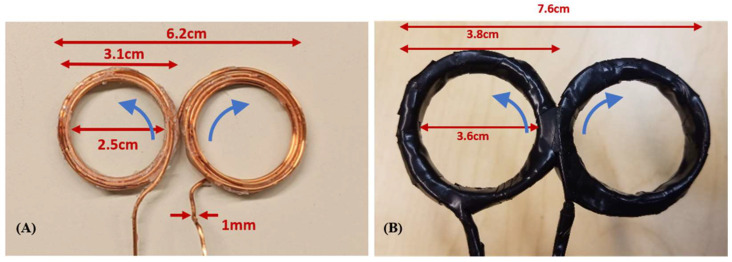
Experimental figure-8 coils. (**A**) Coil A, with 2.5 cm loop inner diameter and N = 9. (**B**) Coil B, with 3.6 cm loop inner diameter and N = 6. Blue arrows show the current direction in the coils.

Experimental Design and Methodology. To provide experimental results corresponding to the design simulations, multiple miniaturized figure-8 coils were prepared, and two are shown in Figure 4. Coil A had a 50 mm inner margin, and the outer margin, taking into account the wire thickness, was 62 mm. It had N = 9 turns in each loop. The coil inductance was measured using an Agilent E4980A LCR meter to be 7.28 µH; a corresponding simulation using the Maxwell ElectroMagnetic simulator yielded 7.83 µH, which is in very good agreement. Coil B had a maximum size of 76 mm; the inner margins of the coil were 72 mm across, and the outer margins were separated by 76 mm. The coil contained N = 6 turns for each loop, and a measured inductance of 3.89 µH. Measurements under pulse excitation were made using a compact rTMS system primarily consisting of a printed circuit board (PCB) with IGBT and diode-based electronics.

**Figure 5:**
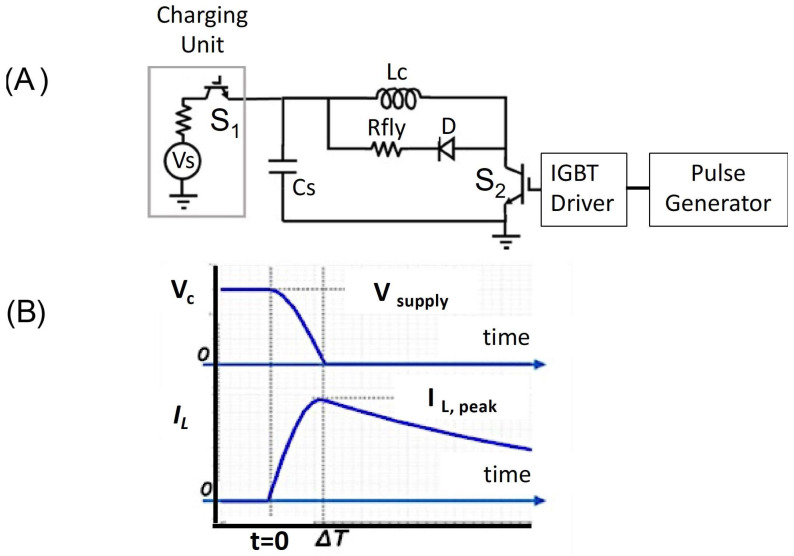
(**A**) Primary circuit design showing IGBTs. (**B**) Simulated waveforms for the main capacitor voltage and the coil current, providing qualitative representations of system behavior.

The design of the circuit was assisted by the use of a transient circuit simulator, ADS from Keysight Technologies. The prototype circuit shown in Figure S1 was implemented on a printed circuit board using a switch S_2_ based on Si IGBTs (model IXXK300N60B3 from IXYS Corp., Chicago, IL, USA) with a maximum voltage of 600 V and a maximum pulse current of 1140 A; four IGBTs were connected in parallel to provide appropriate current handling (see Figure 5A). Diode D was implemented with a parallel combination of Si diodes IDW75D65D1XKSA1-ND from Infineon, Neubiberg, Germany. The IGBTs and diodes were sized to allow a current up to 3.5 KA in short (<100 ms) pulses. Drive voltage to the IGBTs was provided by IX21844N gate drivers from IXYS Corp., Chicago, IL, USA, and turn-on and turn-off times were estimated to be below 0.3 ms. The main discharge capacitor C was a Kemet model C4DE 380 mF with non-inductive winding and a metallized polypropylene film dielectric rated to 400 V. The capacitor used a polymer dielectric to ensure high pulse current carrying capability. The initial voltage V_o_ was varied up to a maximum of 350 V using an external supply, although a separately demonstrated PCB-based boost converter could provide this voltage starting from a 20 V battery and connected to C by a switch S_1_ with controllable timing to allow C to be charged when appropriate.

System monitoring relied on a Hall effect sensor and a Rogowski coil. The Rogowski coil with a current waveform transducer (Power Electronic Measurements, Inc., Nottingham, UK) measured the high-speed current pulse $I_{L}(t)$. The capacitor voltage $V_{C}(t)$ was measured on an oscilloscope. Hall effect magnetic sensors (DRV5056, Texas Instruments, Dallas, TX, USA) were placed at various distances from the center of a coil loop to measure the magnetic field (B-field) strength along the *z*-axis. The x-directed electric field was measured in air as a function of distance from the bottom of the coil. The measurement employed a “pickup” coil consisting of a straight wire segment of length L_s_ = 1.2 cm, oriented along the direction of the electric field, and additional perpendicular wire segments that extended well beyond the region of the induced electric field (and were perpendicular to the induced electric field). The pickup coil was connected to a high-input-impedance oscilloscope to determine the time-dependent coil voltage V(t), from which the electric field E(t) = V(t)/L_s_ was calculated (see Supplementary Material for further detail). The pulsed stimulus current to the coil was provided with the switching inductor/capacitor circuit shown in Figure 5A. A diode-connected current return path was used to produce a monophasic current pulse. The capacitor was fully charged to the supply voltage (V_supply_) and then discharged through the inductor, producing a current pulse with peak current when the capacitor voltage dropped to zero (Figure 5B). For the waveform described here (similar to the waveforms of numerous other systems), the maximum voltage occurred at the beginning of the pulse (corresponding to highest dI/dt) and the maximum current at the time ΔT, when the induced electric field dropped to zero. The current waveform during this period followed approximately sinusoidal behavior for one quarter wave of the period of the LC resonant circuit formed between the coil and the storage capacitor, given by

(4) $$∆T=\frac{\pi }{2}\sqrt{LC}$$

This value of ΔT corresponds to the effective pulse duration for the induced electric field; after this time, the electric field across the coil dropped to zero, and the switch from the coil to the ground was opened (after which the coil current began to decrease through the diode D). The energy stored in the electric field of the capacitor (½ CV^2^) was approximately the same as the energy stored in the magnetic field of the inductor (½ LI^2^) at peak current, with a correction due to the energy ΔE_R_ dissipated in the resistance of the circuit during the time ΔT:

½ CV^2^ = ½ LI^2^ − ΔE_R_ (5)

The value of ΔE_R_ can be related to the magnetic energy for the inductor E_L_, according to

ΔE_R_/E_L_ = R ΔT/L (6)

where L/R is the time constant associated with the coil inductance L and the network resistance R. In our network, the value of ΔE/El was below 30%, with resistance contributions from the coil, the capacitor, and the IGBT switch. The peak current can be approximated by:

(7) $$I_{peak}=\frac{V_{supply}}{\sqrt{L/C}}$$

However, in practice, the value is reduced slightly by resistance effects.

From Equation (7), the inductor current increases with supply voltage and capacitance, but decreases with the coil inductance. There are limitations on the maximum supply voltage (V_supply_) and capacitor size (C) because of the portability, safety, and low power operation of the system.

## 3. Results

Measurements of coils A and B were carried out with slightly different switching hardware, in accordance with the coil inductance. The capacitor C had a value of 380 µF. A comparison of maximum operating voltage and current is shown in Table 3 from measurements of coils A and B, in addition to the pulse duration as expected from Equation (4), R_ch_ for the switches, and the relationship with L/γΔT. The table shows the values of V_max_ and I_max_ for experimental cases, which allows one to calculate the characteristic resistance Rch of the switch for these cases. The values found are in good agreement with the values that were calculated from Equation (2) according to the choice of L and pulse width used.

**Figure 6:**
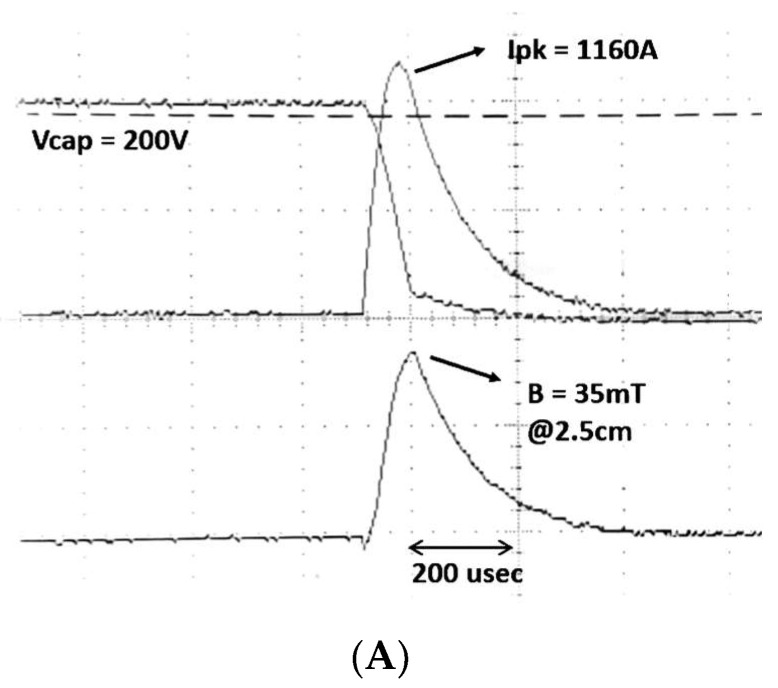

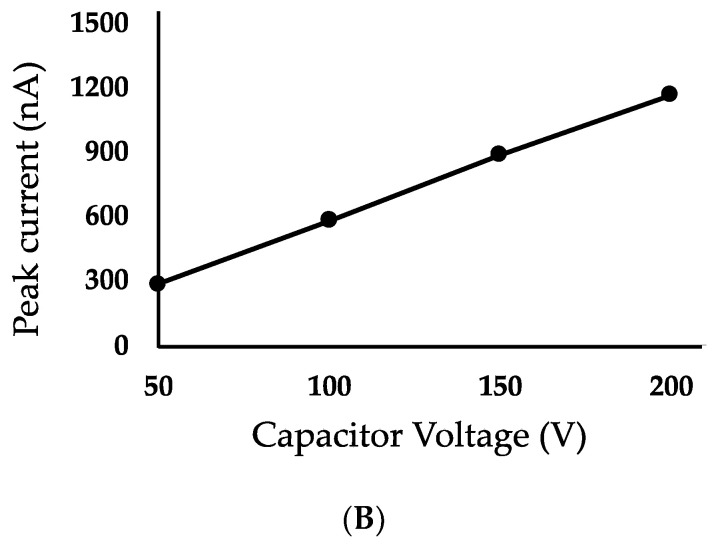
Measured peak current versus voltage (coil A). (**A**) Oscilloscope trace of an experimental pulse. (**B**) Measured peak current vs. initial capacitor voltage.

The measured voltage is shown in Figure 6A, along with the current and the B-field from our test apparatus. This figure illustrates the good coresppondence with Figure 5B. Figure 6B shows the linear relationship between the voltage applied to the capacitor and the measured peak current.

**Figure 7:**
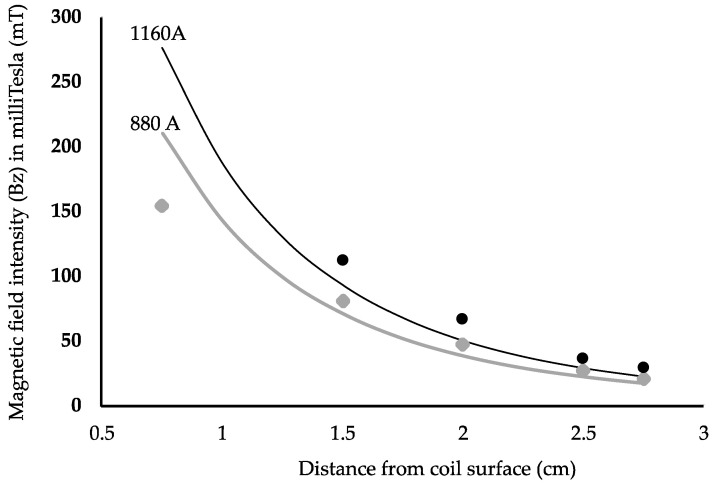
COMSOL simulation results and experimental measurement comparison of the device B-field in the z-direction for a 62 × 31 mm coil (coil A). Simulated and measured data are shown for I = 880 A, with the lower COMSOL simulation curve in gray, and I = 1160 A, with the upper COMSOL simulation curve in black. The gray triangles denote experimentally measured values at 880 A, while the filled black circles indicate experimentally measured values at 1160 A.

The B-field simulation and experimental results were compared to ensure consistency between the simulation and the experiments. The results are shown in Figure 7 for the 62 mm coil A with electrical current peak values of 880 A and 1160 A. The current pulse width was 83 µs. Figure 7 compares the magnetic flux density at various points along the *z*-axis, i.e., distance or depth from the center of a coil loop, for simulated and measured values.

**Figure 8:**
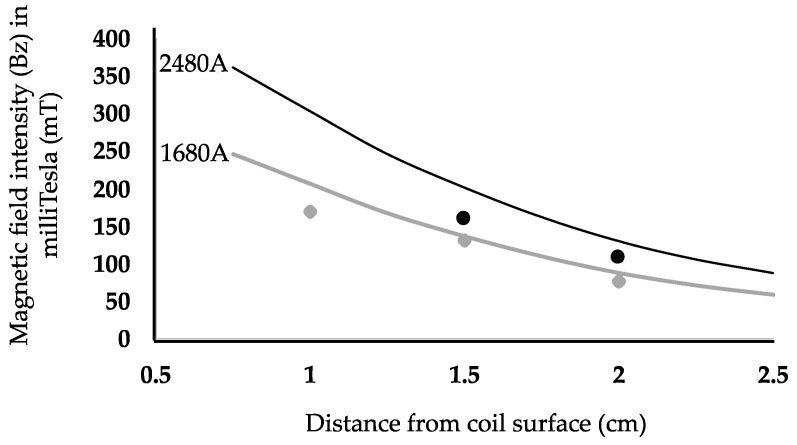
COMSOL simulation results and experimental measurements of the B-field in the z-direction in a 76 × 38 mm coil (coil B) for I = 1680 A and I = 2480 A. Simulated and measured data are shown for I = 1680 A, with the lower COMSOL simulation curve in gray, and I = 2480 A, with the upper COMSOL simulation curve in black. The gray triangles denote experimentally measured values at 1680 A, while the filled black circles indicate experimentally measured values at 2480 A.

**Figure 9:**
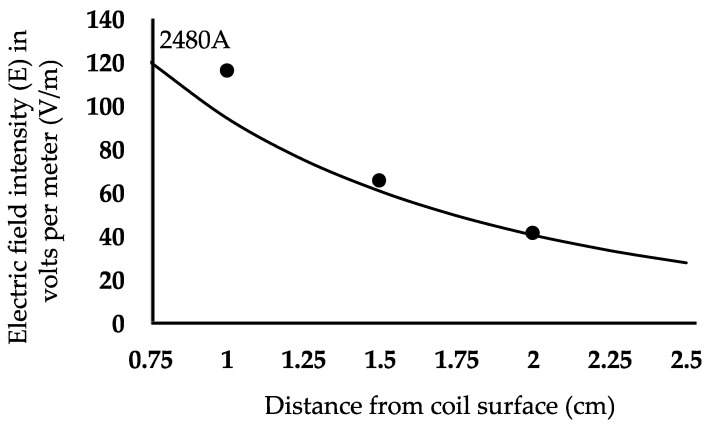
Graph showing E-field data for a COMSOL simulation versus experimental measurement using a 76 × 38 mm (12.6 g) figure-8 coil (coil B) with I = 2480 A. The solid line represents the simulation plot, while the filled black circles denote experimentally measured values.

**Figure 10:**
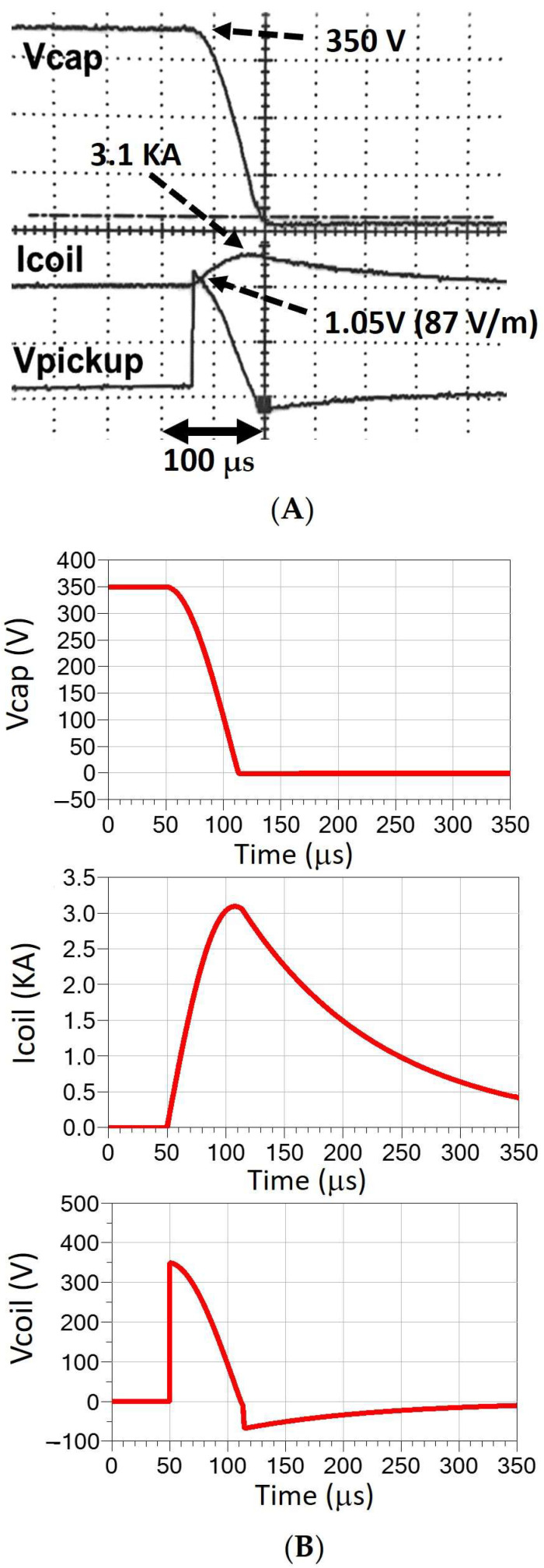
(**A**) Oscilloscope measurement of V_cap_ (350 V), I_coil_ (3.1 KA), and V_pickup_ (1.05 V) corresponding to E = 87 V/m at 1.5 cm, for a 76 × 38 mm figure-8 coil (coil B). (**B**) Simulated values of the waveforms. Voltage across the coil is shown, related to Vpickup by a depth-dependent proportionality factor.

Coil B was tested using peak currents of 1680 A and 2480 A for capacitor voltages of 200 V and 300 V, respectively. The pulse width was 55–60 µs. We measured both the B- and E-fields in air. We also performed COMSOL simulations for B-fields and E-fields using the head model. Comparisons between the simulations and the experimental results are shown in Figure 8 for the B-field and in Figure 9 for the E-field. In order to test the maximum E field that could be generated with coil B within the V_max_ and I_max_ limitations of the switch used, we also measured the system performance with V_cap_ = 350 V and resulting I_max_ = 3.1 kA. Figure 10A shows pulses for capacitor voltage (V_cap_), coil current (I_coil_), and pick up coil voltage (V_pickup_), representing the monophasic pulse. The corresponding E field at 1.5 cm depth was 87 V/m. Figure 10B shows the simulated values of the waveforms, which can be directly related to measured values.

With repetitive pulsing, the system was demonstrated in operation at 10 Hz, which is widely used in rTMS clinical practice. Overall limits on the maximum pulse rate and maximum power were set by potential heating of the coil. With the monophasic waveform used here, the energy stored in the capacitor for each pulse dissipated within the system components. The power dissipation in our circuit took place primarily in the flyback resistor (labeled Rfly in Figure 5A). However, the resistance of the coil together with the large coil current induced coil heating, both during the charging period when the switch was closed and later, during the discharge period, when current flowed through the flyback path.

While most of the pulse energy is dissipated in the fly-back resistor, the integrated energy input per pulse dissipated in the coil can amount to 1 to 5 J at high intensity. When the system is operated at full power, the energy per pulse is ½ CV^2^ = 23 joules. This energy is partially conducted away by convection and by the coil leads, but it also leads to a rise in coil temperature. Preliminary measurement of the rise in temperature at 10 Hz pulsing for short times and moderate intensities showed a temperature rise below 8 degrees. This finding indicates that future research and development of miniaturized coils along the lines delineated by the present study will need to be designed and optimized in terms of thermal management to be able to sustain very long periods of high-intensity 10 Hz pulse generation.

## 4. Discussion

The demonstrated E field for coil B at 1.5 cm is within the range generally considered to be useful in therapeutic applications. With the constraints used in the measurement (350 V and 3.1 kA), it is also in the range of, albeit lower than, the fields obtainable with commercial coils and clinical rTMS systems. It is important to note that power consumption depends on several factors, including the rTMS pulse train pattern, pulse width, and frequency. As a result, reporting a number for power consumption would not be accurate or beneficial. Importantly, Table 4 shows a comparison between our system at 350 V and several major representative commercially available systems at 1.3–3 kV. The electric fields generated by our rTMS platform are well within the therapeutically relevant range. The maximum B fields are significantly smaller, however. This is in keeping with design guidelines, since the B field is known to be less directly relevant for therapy. It is noteworthy that our system operated with a maximum voltage of 350 V, while commercial systems use substantially higher voltages, e.g., 2800 V for the MagStim clinical rTMS machine. Significantly, the E-fields, at an approximate cortical depth of 1.5 cm, are well above the limit of 28 V/m, which is the minimum intensity needed to induce a neuronal action potential [42,43].

**Figure 11:**
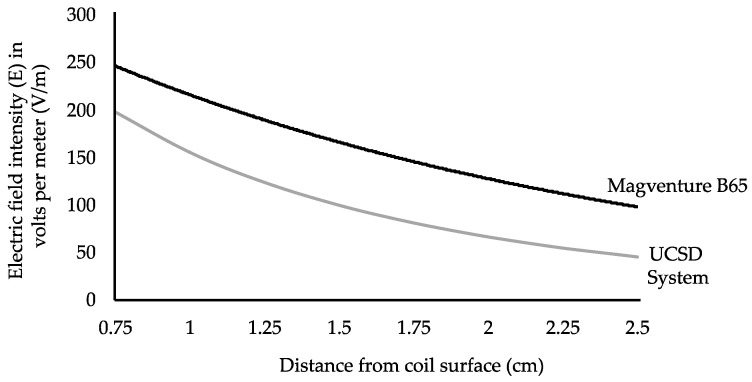
Graph showing the E-field for the MagVenture clinical rTMS R30 device driving a 2 kg 172 × 92 mm B65 figure-8 coil at peak power, 1.4 T, upper black line, and the simulation for coil B (76 × 38 mm) coupled to our driving circuit, verified by experimental measurments at 1.5 cm from the center of the coil surface.

Figure 11 contrasts the calculated E field vs. the depth profile for coil B under the maximum measured conditions, with the E-fields for the MagVenture clinical rTMS R30 device driving a B65 figure-8 coil at peak power (1.4 T) (R30/B65 data provided courtesy of MagVenture and used with permission). The miniaturized coil and rTMS electronics reported here attained 52% of the peak electric field produced by the commercial system at 1.5 cm (168 V/m).

The roll-off of the electric field with depth was somewhat steeper for the miniaturized coil, as expected from the scaling laws, since the coil dimensions of 76 mm × 38 mm were scaled from the MagVenture 172 mm × 92 mm coil by a factor of 0.44×. The miniaturized coil thus did not reach as far into the brain as is possible with the commercial coil. The maximum effective depth possible with the miniaturized coils is a function of the driving electronics, including the maximum voltage that is used in the system and the characteristic impedance R_ch_ of the switches.

The central criteria guiding this work were coil size and weight. The two experimental figure-8 coils were significantly smaller and much lighter than the presently available commercial clinical rTMS coils. The 76 mm × 38 mm coil weighed only 12.6 g (0.4 oz), which contrasts markedly with the weight of 1.8–3.9 kg (4–8.6 lbs) of a clinical rTMS head coil.

We used the COMSOL head-coil model to predict E fields without detailed consideration of the effects of sulci and specific nerve tissue geometries, which yielded values that can be directly compared to most reported results [48,49,50,51,52,53,54,55]. These predicted values were then compared to E field measurements in air using our experimental set up. This is a limitation of this study, as the head model simulations involved different electrical properties than measurements in air did. This limitation was accepted in the interest of throughput and simplicity, i.e., to avoid the construction of a complex head phantom with imbedded electrodes.

Neurobiological considerations include the fact that operation of the board was simplified via the application of a monophasic pulse only, not a biphasic pulse. It is assumed that monophasic and biphasic pulses may elicit similar neurophysiological effects, but relatively little work has been conducted in this arena. From a practical design standpoint, a biphasic pulse may offer the significant advantage of allowing for energy recovery, but this entails extensive additional circuitry. Therefore, we opted for monophasic pulse generation to achieve higher portability. We regarded this as an acceptable trade-off, as the physiologically critical parameter is pulse rise time and slope (dB/dT). Moreover, both monophasic and biphasic pulses can trigger neurophysiological effects, and as alluded to above, any clinically relevant difference between the two remains to be fully resolved.

**Figure 12:**
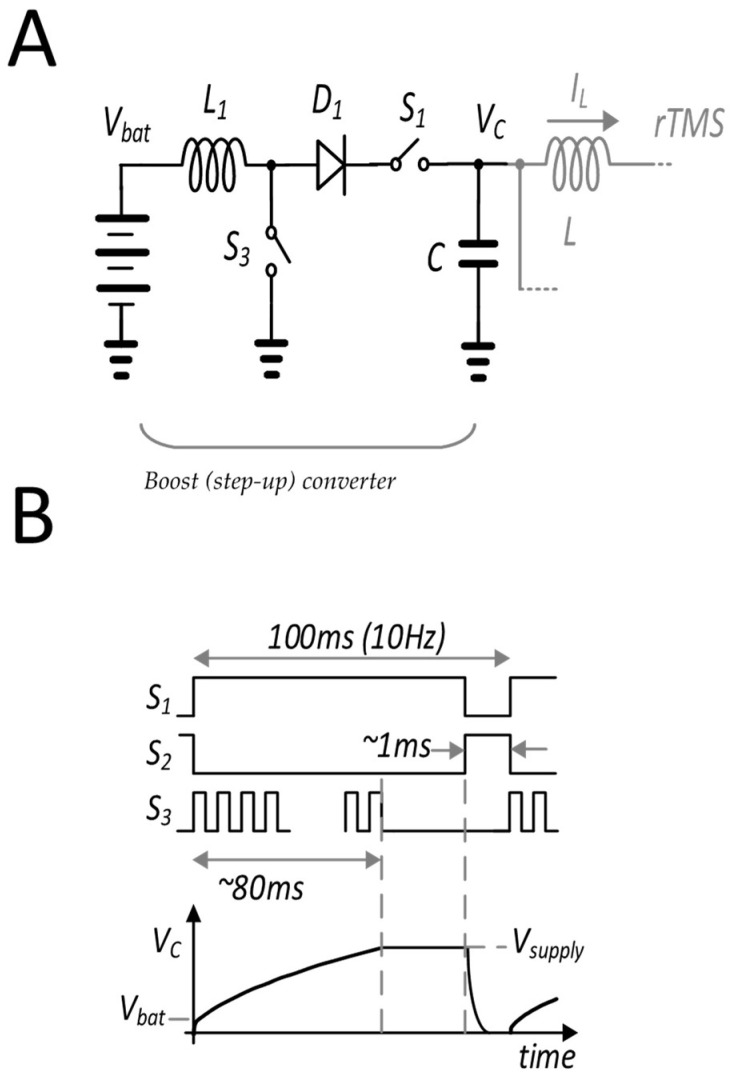
Boost converter to increase power efficiency. The boost converter design is shown in (**A**); the boost step-up is depicted graphically in (**B**).

An additional important consideration for the system’s implementation is the maximum voltage needed. With the designed coils, the clinically relevant electric fields can be achieved with a power supply value of 350 V, while clinical systems described in Table 3 require 1200 V or higher. The use of such high voltages creates significant requirements for electrical insulation and safety interlocks, and leads to larger and more costly electrical components such as capacitors and switches. Accordingly, with their significantly lower weight, size, voltage, and power requirements compared with rTMS systems in current clinical use, the miniaturized systems described here could have potential for mobile or portable operation using battery-based power sources. Certainly, achieving portability at 350 V is a challenge, and we note in this context that a boost converter is available in our system, although not used in all of the coil-related experiments, to make improved use of available power and to provide the rTMS pulses using a 20 V battery, as shown in Figure 12.

As detailed earlier, the main advantage of the system developed here in relation to existing commerical rTMS systems is its reduced size and weight, both for the head coil and the driving circuit. Importantly, the small coil size compared to commercially available coils may also allow for improved focality. This is evident in Table 1, where focality is predicted through FEM for different coil sizes. Since our simulation results are in close accordance with experimental measurements for both electric and magnetic fields (Figure 7, Figure 8 and Figure 9), it is reasonable to conclude that the focality values deduced from the simulation are representative of the focality performance of the coil. However, we recognize that design variations may be implemented to optimize focality for specific clinical and experimental applications.

In the system we describe herein, the pulses are 100 μs long, repeated at 10 Hz for 2–3 sessions which are each 10 min long. With three sessions, the total integrated duty time is only 1.75 s, as the system is not continuously generating E-fields in an uninterrupted way. However, the E-fields produced by the present system are smaller than the ones produced by full -sized existing products, as demonstrated in Table 4 and Figure 11. This could place some limits on the therapeutic applicability of the miniaturized system, although the E-fields produced here are still generally clinically relevant [26,27,28,42,43]. Moreover, the energy required for each pulse in our system is significantly less than that for existing products due to the peak voltage being only a fraction (0.1–0.3) of most commercially available systems, and some examples are listed in Table 4. This may support operation using a rechargeable battery and may reduce heating.

## 5. Conclusions

This report identifies novel design criteria for a miniaturized head coil and driving circuit to be used within a compact, portable rTMS system. Scaling characteristics are described, and the importance of impedance matching between the inductance and the switching electronics is identified. E fields measured with a coil with scaled dimensions operating at 350 V and 3.1 KA reached 87 V/m at 1.5 cm. This field intensity is within the range considered to be therapeutically useful, suggesting that the prototype can be adapted for clinical use [26,27,28,42,43]. The concepts and results presented here illustrate key design considerations and hold promise for rTMS system miniaturization in order to advance rTMS treatment to a broad array of applications and beyond the confines of a large medical facility. Importantly, a miniaturized head coil may form the core of a wearable ambulatory device to offer greater opportunities for the rTMS-based management of a range of neuropsychiatric and addictive disorders.

## Figures and Tables

**Table 1:** Physico-electromagnetic properties of head layers [41].

Head Layer	Thickness (mm)	Mean Conductivity (S/m)	Relative Permittivity (F/m)
Skin—Scalp	5	0.465	1.2 × 10^2^ε_0_
Bone—Skull	10	0.010	0.8 × 10^2^ε_0_
Cerebrospinal Fluid	3	1.654	0.6 × 10^2^ε_0_
Gray Matter (GM)	5	0.276	1.2 × 10^2^ε_0_
White Matter (WM)	77	0.126	1.2 × 10^2^ε_0_

**Table 2:** Coil size versus performance metrics.

Coil Size L (mm)	$E_{max}$(V/m)	$d_{1/2}$ (m)	$S_{1/2}$ (m^2^)
50	286.79	4.00 × 10^−3^	1.04 × 10^−4^
100	384.66	5.41 × 10^−3^	2.59 × 10^−4^
150	431.96	6.5 × 10^−3^	3.91 × 10^−4^

**Table 3:** Relationships between coil and switch characteristics.

Coil	A	B
L (µH)	7.28	3.89
ΔT (µs)	83	60
V_max_ (V)	300	350
I_max_ (A)	2100	3000
R_ch_ (Ω)	0.143	0.117
L/γΔT (Ω)	0.138	0.10

**Table 4:** Comparison of this work with commercial systems.

rTMS Coil—Driving Circuit System	Peak Voltage (V)	Maximum B-Field (T)	Maximum E-Field at 1.5 cm (V/m)	Coil Weight (g)	Coil Dimension (mm)
Coil A (this work)	200	0.47	65	12.6	62 × 31
Coil B (this work)	350	0.54	87	12.6	76 × 38
Remed Brain-StimA	1200	2.5	-	2000	160 × 80
Magventure B65	1850	1.4	168	1800	172 × 92
Magstim D70/200^2^	2800	0.92	116	1760	180 × 90

## Data Availability

The authors would be pleased to provide the original data, requests should be conveyed to the senior, corresponding author.

## Supplementary Materials

The following supporting information can be downloaded at: https://www.mdpi.com/article/10.3390/s24051584/s1, Figure S1: Experimental setup; Figure S2: E-field measurement; Table S1: The mesh statistics parameters for coil size L = 150 mm; Table S2: The solver parameters for field calculations; Idealized scaling of electric and magnetic fields for figure-8 coils.
