# Supplementary material for: Design and Validation of Miniaturized Repetitive Transcranial Magnetic Stimulation (rTMS) Head Coils
Source: Sensors (Basel). 2024 Feb 29;24(5):1584. doi: 10.3390/s24051584 (PMC10934069; doi:10.3390/s24051584)
Supplement: Supplementary file 1 [file sensors-24-01584-s001.zip › sensors-2734523-supplementary.pdf]

sensors MDPI

Supplementary Materials

COMSOL Simulation Setup

A finite element model of the head and the rTMS coil was developed using AC/DC module in COMSOL Multiphysics®. An infinite element domain was used to set the boundary conditions. The total number of mesh vertices was in the range of 9527 to 10974 depending on the coil size. The details of mesh statistics are included in Table S1 for coil size L = 150 mm. The details of the solver parameters are included in Table S2.

Table S1. The mesh statistics parameters for coil size L = 150 mm.

Parameter	Value
Mesh vertices	10974
Tetrahedra	62957
Triangles	9151
Edge elements	1269
Vertex elements	100
Minimum element quality	0.009355

Table S2. The solver parameters for field calculations.

Parameter	Value
Nonlinear method	Automatic (Newton)
Initial damping factor	1
Minimum damping factor	1E-4
Restriction for step-size update	10
Restriction for step-size increase	1
Recovery damping factor	0.75
Termination technique	Tolerance
Maximum number of iterations	25
Tolerance factor	1
Termination criterion	Solution or residual
Residual factor	1000

Experimental Setup

The prototype test system, along with the measurement setup is shown in Figure S1. A test circular coil with well-established characteristics is used to validate the measurement system, which employs a Rogowski coil for pulsed current measurements and a high-speed Hall sensor array to document magnetic fields vs position along the axis of the coil.

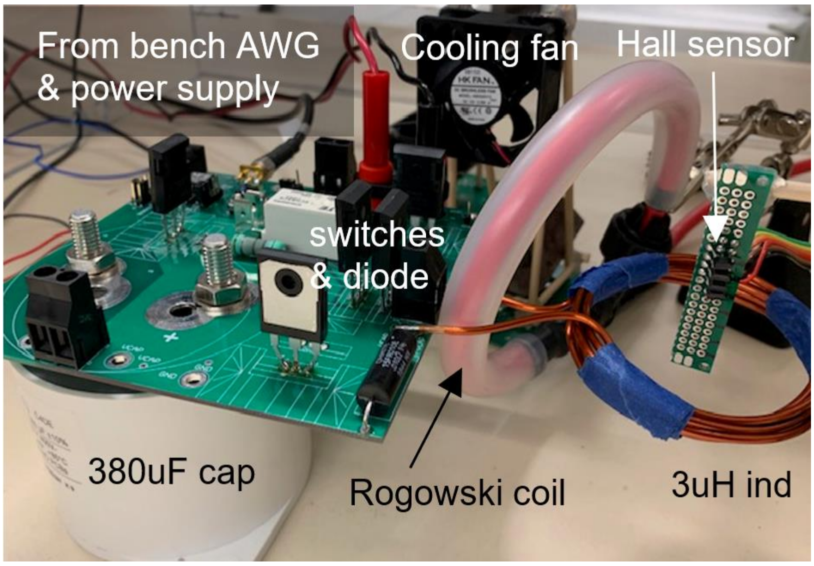

Figure S1. The prototype test system and the field measurement setup.

E-Field Measurement

The probe used to measure the electric field is shown in Figure S2. The relevant E-field is in the y direction. The pickup coil extends only in the y-z plane, with dimensions $\Delta y = 0.8\ cm$ and $\Delta z = 25\ cm$.

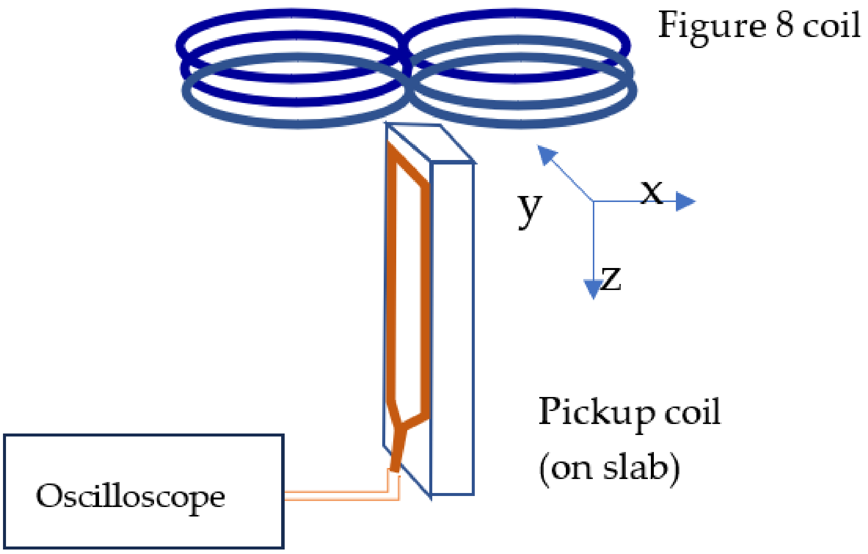

Figure S2. E-field measurement setup. The pickup coil is in y-z place, while the figure 8 coil is in x-y plane.

A key issue is that the coil produces no electric field along the vertical (z-directed) wires. The coil current is entirely in the x-y plane, and as a result so is the magnetic vector potential A. The E-field is aligned along $\vec{A}$.

$$\vec{A} = \frac{\mu}{4\pi}\int \frac{I(\overrightarrow{r'})}{|\vec{r}-\overrightarrow{r'}|}d\overrightarrow{r'} \qquad \vec{E} = \frac{\partial \vec{A}}{\partial t} \tag{S1}$$

Simulations indicate that the electric field is approximately constant over distances of 1 cm at depths of 1 or 2 cm from the coil, although the measured values are an under-estimate of the peak field. $E_y = V_{pickup}/L_{pickup}$; $L_{pickup} = 0.8$ cm

Idealized scaling of electric and magnetic fields for figure-8 coils

We consider an idealized figure-8 coil with current I carried in a 1-dimensional wire consisting of two circular sections, displaced in the x direction by a distance equal to the coil diameters. The fields of greatest interest lie underneath the intersection of the two circular portions, at x=y=0 at different depths z. The electric field reaches its maximum at this position. The fields may be calculated with the use of the vector potential A, using equations in S.1 along with the following equation:

$$\vec{B} = \nabla \times \vec{A} \tag{S2}$$

where μ is permeability and I is the electric current. For the figure 8 coil, at x=0, y=0, A is exclusively x-directed, and can be written as a function of the parameter (z/R) and no other dimensional values, where R is the radius of the coil, and z is the vertical distance from the coil center. An explicit relationship for A_x (x=0, y=0, z) is

$$A_x(z) = \frac{\mu I}{4\pi}\int_0^{2\pi} \frac{cos\theta d\theta}{\sqrt{\left(\frac{z}{R}\right)^2 + 2(1 + cos\theta)}} \tag{3}$$

Calculated E_x and B_y fields for different single loop coils of radius R, at various depths z from the coil are shown in Figure 1. Values of E are proportional to dI/dt, while the values of B are proportional to I; in the figure, the fields are normalized to their values for z=2 cm and R=10 cm.
